# Pharmacokinetic Interactions between Cardiovascular Medicines and Plant Products

**DOI:** 10.1155/2019/9402781

**Published:** 2019-09-02

**Authors:** Irina-Iuliana Costache, Anca Miron, Monica Hăncianu, Viviana Aursulesei, Alexandru Dan Costache, Ana Clara Aprotosoaie

**Affiliations:** ^1^Faculty of Medicine, Grigore T.Popa University of Medicine and Pharmacy Iasi, 700115 Iasi, Romania; ^2^“Sf. Spiridon” University Hospital, 700111 Iasi, Romania; ^3^Faculty of Pharmacy, Grigore T.Popa University of Medicine and Pharmacy Iasi, 700115 Iasi, Romania

## Abstract

The growing use of plant products among patients with cardiovascular pharmacotherapy raises the concerns about their potential interactions with conventional cardiovascular medicines. Plant products can influence pharmacokinetics or/and pharmacological activity of coadministered drugs and some of these interactions may lead to unexpected clinical outcomes. Numerous studies and case reports showed various pharmacokinetic interactions that are characterized by a high degree of unpredictability. This review highlights the pharmacokinetic clinically relevant interactions between major conventional cardiovascular medicines and plant products with an emphasis on their putative mechanisms, drawbacks of herbal products use, and the perspectives for further well-designed studies.

## 1. Introduction

The use of herbal products and dietary supplements with botanical extracts has increased tremendously across the world over the past two decades. WHO estimated that about 80% of the world population in developing countries rely on the plant products as major agents for primary health care and 33% of the population in developed countries use various herbal products and dietary supplements [1]. Herbal medicine or green medicine is perceveid as a more balanced therapeutic approach. It is generally considered a natural, safe, and inexpensive alternative to healing and to promote a healthier living style, the latter being the major reason for the plants use in the developed countries.

The presence of this increased interest and the variety of products on the global market raises the issue of the effects that can occur from the association with various medicines or even other herbal products [2]. Supplementing pharmacotherapy with herbal products has attracted much attention. It is estimated that 20-35% of prescription drugs users also use plant products [3]. More than 50% of patients with chronic diseases or cancer use dietary supplements, and almost 1/5 of patients use prescribed medicines and supplements concomitantly. The potential risks associated with combined use of drug and dietary supplements are poorly understood, especially by patients, but there is still limited knowledge among specialists as well [4]. The most important clinical consequence of the combined use of herbal products and medicines is drug interaction as revealed by numerous case reports, controlled pharmacokinetic and clinical studies over the last 20 years [2].

Plant products are usually complex mixtures of various compounds such as phenolics, phenolic glycosides, alkaloids, peptides, polysaccharides, resins, essential oils that impart diverse bioactivities. The chemical composition and biological activity of phytopreparations are strongly related to the quality of plant material and its processing and formulation. The complexity and variability of plant products increase the risk of drug interactions. In addition, patients' positive perception on herbal products and their widespread acceptance cause them not to inform their physicians when they use these preparations (“*don't ask - don't tell*”), which also complicates the picture of the assessment or predictability of these drug-plant interactions. Cardiovascular medicines, oncology, immunosuppressant, and CNS drugs are the most involved in the interactions with plant products [3, 5]. 

**Figure 1:**
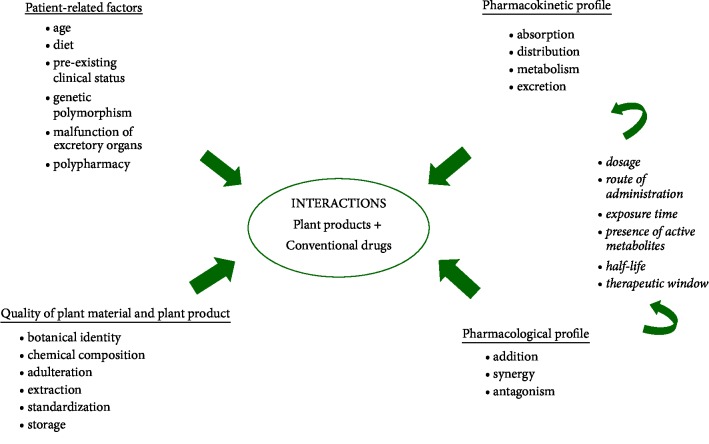
The important risk factors that influence the occurrence of interactions between plant products and conventional drugs.

166 botanical extracts and various plant products are responsible for about 60% of the total interactions reported in articles between 2000 and2010. The most documented products in terms of reported interactions are:* Hypericum perforatum* (St. John's wort),* Ginkgo biloba,* and grapefruit juice [4]. Thus, it is estimated that grapefruit juice interacts with 85 medicines and half of these interactions are potentially dangerous. Interactions may be mainly pharmacokinetic or pharmacodynamic. Concomitant use of plant products or dietary supplements with conventional drugs or their association can change drugs systemic exposure and their pharmacological effect and influence the therapeutic efficiency and the risk of drug toxicity. The clinical significance of these interactions is influenced by several factors such as drug and patient-related factors but also plant products quality (Figure 1). In case of medicines with a narrow therapeutic window, even a modest increase of plasma concentrations can be translated into serious adverse effects [6].

The aim of this paper is to provide an overview of most clinical pharmacokinetic relevant interactions of major pharmacological classes of cardiovascular drugs with plant products, their mechanisms, phytochemicals that are involved, and drawbacks of herbal preparations use. Also, some future perspectives for investigating the interactions between plant products and conventional drugs are presented.

## 2. Pharmacokinetic Interactions

These are most documented interactions between plant products and drugs/cardiovascular drugs. Plant products can alter absorption, distribution, metabolism and excretion (ADME) of drugs. Pharmacokinetic interactions involve primarily the up- or down-regulation of the human cytochrome P450 (CYP) enzyme's activity and drug transporters including organic anion and cation transporters, as well as nuclear pregnane-X receptor [2]. Consequently, the change in the oral bioavailability of drugs and their metabolic clearance occurs, resulting in therapeutic failure, toxic or beneficial responses.

Induction or inhibition of the CYP450 activity is one of the most important pharmacokinetic interaction. CYP450 is a family of enzymes involved in the oxidative metabolism (phase I) of most drugs in clinical practice [5]. The major drug-metabolising enzymes are CYP1A2, CYP2C8 /2C9/2C19, CYP2D6, and CYP3A4. CYP3A4 is the isoenzyme with the highest level of expression in the gut and liver and with the lowest substrate specificity [7]. CYP3A4 is the main metabolic pathway of over 60% of the drugs currently used, representing 35% and 80%, respectively, of the CYPs expressed in the liver and the small intestine, respectively [8]. The induction of metabolic enzymes results in an increase in systemic clearance and in therapeutic failure. Metabolic enzymes inhibition may manifest clinically by an increase in systemic exposure of drug with phenomena of overdosing and increased toxicity [5]. CYPs induction is slow and a regulated process that takes time to achieve a higher steady-state enzyme levels, while the inhibition of CYPs is an almost immediate response. CYPs induction occurs through receptor-mediated mechanisms that cause an increase in gene transcription. The transcriptional activation is mediated by nuclear factors that act as transcription factors such as: aromatic hydrocarbon receptor (AhR), constitutive androstan receptor (CAR) and pregnane X receptor (PXR). Induction and inhibition phenomena depend on exposure time, repeated exposures can lead to induction, and a single dose can cause inhibition [1]. Inhibition of CYPs is the most common mechanism. Enzymatic inhibition can be classified as reversible (competitive and non-competitive) and time-dependent. Unlike reversible inhibition, time-dependent inhibition may persist even after the disappearance of the agent causing the interaction because the recovery of enzyme activity requires* de novo* synthesis of proteins [5].

Alongside the CYP450 system, uridine-diphosphate (UDP)-glucuronosyltransferases (UGTs) are major enzymes responsible to the detoxification of a wide range of xenobiotics and endobiotics. They are involved in the main phase II reaction (glucuronidation), one of the most important clearance pathways in humans However, the effects of plant products on glucuronidation and their interactions with drugs that are UGTs substrates have not been sufficiently studied. Based on* in vitro* and animal studies, some flavonoid-containing plants such as cranberry (*Vaccinium macrocarpon*),* Ginkgo biloba*, grape seed (*Vitis vinifera*), green tea (*Thea sinensis*), hawthorn (*Crataegus oxyacantha*), milk thistle (*Silybum marianum*), noni (*Morinda citrifolia*), soybean (*Glycine max*), valerian (*Valeriana officinalis*), and St. John's wort (*Hypericum perforatum*) as well as fatty acids may modulate UGTs function but the clinical consequences of these effects are poorly understood. Only three clinical trials have investigated the effects of plant extracts on pharmacokinetics of drugs that are metabolized primarily by UGTs, namely, the interactions between garlic and acetaminophen, milk thistle and irinotecan, and American ginseng and zidovudine, respectively [9, 10]. Among cardiovascular medicines, carvedilol, a non-selective beta-blocker, is mainly cleared by glucuronidation via UGT1A1, UGT2B4 and UGT2B7 enzymes intervention, but clinical data on carvedilol and plant products/phytochemicals interactions mediated by UGTs mechanisms, have not been reported to date [9, 11]. Further investigations are needed to evaluate the potential of the plant products to interact with UGTs-mediated drug metabolism and to determine the clinical significance of these interactions.The effects of dietary intervention on UGTs activity in humans is also important and it can include in further research. It has been shown that citrus fruits consumption may increase UGT1A1 enzyme activity among women with the 7/7 genotype (UGT1A1*∗*28 variant alleles) leading to the alteration of both drugs and carcinogens metabolism [10, 12].

The pharmacokinetic profile of drugs can also be modified by altering the functions and expression of transport proteins from the ATP-binding cassette (ABC) family and solute carrier (SLC) categories. The ATP-binding cassette (ABC) family includes P-glycoprotein (P-gp), multidrug resistance protein (MRPs) and breast cancer resistance protein (BCRP) and it affects the efflux of their substrates. SLC family, including organic anion transporters (OATs), organic cation transporters (OCTs) and anion organic transporting polypeptides (OATPs), mediates their uptake. They are involved in oral and kidney absorption and hepatobiliary availability of drugs. From a clinical point of view, transporters modulation may be manifested as either an increase or decrease in systemic availability depending on the transport direction (efflux/influx) and the location (apical/canalicular, basolateral/sinusoidal) [5]. ABC binding cassette transporters, OATPs, OATs and OCTs are the major carriers involved in the efflux and influx of cardiovascular drugs [13].

Depending on the characteristic pharmacokinetic profile, cardiovascular drugs of different pharmacological categories (beta-blockers, calcium channel blockers, positive inotropes, antiarrhythmics, oral anticoagulants, and statins) can interact with plant based products. The most important reported interactions, having major clinical impact, are presented in Table 1.

### 2.1. Beta-Blockers

Beta-blocking agents are used in the management of cardiovascular disorders that include hypertension, ischaemic heart diseases, arrhythmias, congestive heart failure, and for the prevention of myocardial infarction [14]. They differ highly in their pharmacokinetic properties, which contributes to a great variation in interactions with plant products and their unpredictability. Mostly, talinolol was investigated. It has an oral bioavailability of about 55%, a minimal affinity for CYP3A4, being negligibly metabolised, but it is a substrate for P-gp, MRP2 and OATP transporters [15]; it is therefore used as a model substrate for assessing the role of P-gp in triggering plant-drug and drug-drug interactions.

The administration of an oral ginkgo monodose (120 mg) does not affect the pharmacokinetics of talinolol, but repeated ingestion (14 days) of the* Ginkgo *extract (360 mg/day) enhances talinolol exposure [increases in maximum plasma concentrations by 22-25% and the area under the concentration-time curve (AUC) by 34-36%], by inhibiting P-gp activity. Similarly, the* Schisandra chinensis* extract (300 mg×2/day, 14 days) in healthy volunteers increases the talinolol plasma concentration by 51%; also, it increases the AUC value by 47% and half-life (t_1/2_) by 7%. The mechanism is also based on P-gp inhibition. Patients using* Schisandra* or* Ginkgo* extracts may require dose adjustments of coadministered medicines that are substrates of P-glycoprotein [1, 16]

Curcumin (300 mg/day), the main component of turmeric (*Curcuma longa*) reduces significantly maximum serum concentration (C_max_) and AUC and it increases 1.5 times the talinolol clearance (single dose administration), effects mediated through up-regulation of expression of MDR1 mRNA and function of P-gp.* In vitro*, low concentrations of curcumin (0.5-1 *μ*M) induce P-gp by stimulating P-gp ATPase activity [17]. On the contrary, long-term coadministration of curcumin (1000 mg/day, 14 days) increases the bioavailability of talinolol in subjects with ABCB1 C3435T genotypes, probably through reduction in its excretion via down-regulation of intestinal P-gp (Table 1). Discrepancies between the studies could be explained by different dosages and durate of use, but also by the intervention of genetic polymorphism [18].

St. John's wort reduces the bioavailability of orally and systemically administered talinolol, with a 93% increase in oral clearance and a 31% reduction in AUC. The effects are due to the increase in P-gp levels in the duodenal mucosa and in MDR-1 mRNA [1].

The systemic availability of talinolol and other beta-blockers (atenolol, celiprolol, acebutolol) is much diminished (between 20% and more than 80%) in cointake of grapefruit or orange juices (300-600 mL) 4 hours before or after drug administration, which raises the issue of an inappropriate action of the drug. In the case of celiprolol, its bioavailability decreases significantly when combined with orange juice (C_max_ and AUC decrease by 89% and 83%, respectively), the interaction presenting clinical relevance [19]. The mentioned beta-blockers are OATP1A2 substrates, an uptake transporter expressed in all important organs, mainly on the apical surface of enterocytes and cholangiocytes. Non-metabolized hydrophilic drugs (atenolol, celiprolol) are more affected than the metabolised lipophilic ones (acebutolol). Increased polarity and unmodified excretion are more affected by uptake transport than passive diffusion in the case of intestinal absorption of medicines. Consequently, OATP1A substrates that are mainly eliminated through the kidneys in unchanged form (as it is the case with sotalol) are more likely to undergo a significant reduction in oral bioavailability in combination with grapefruit or orange juices, requiring medicine dose adjustment [20].

The bioavailability of nadolol (OATP1A2 substrate and limited metabolic clearance) decreases significantly after pretreatment for 14 days with a green tea product (700 mL/day) (C_max_ and AUC decrease by 85%). The inhibition of OATP1A2-mediated uptake may be a plausible mechanism (Table 1).* In vitro*, green tea extract and the main catechins [catechin, epigallocatechin 3-gallate (EGCG)] inhibit the activity of the OATP1A2, OATP1B1 and OATP2B1 transporters. The amount of catechins in the product tested in the clinical trial was 2-5 times higher than other typical green tea products (1.54 mg/mL versus 0.25-0.52 mg/mL) [19].

### 2.2. Calcium Channel Blockers (CCBs)

CCBs are valuable agents in the treatment of angina, systemic hypertension, and supraventricular arrhythmias. They undergo a significant first-pass metabolism in the gut and liver being mainly substrates of CYP3A4, and verapamil and diltiazem produce active metabolites. Their oral bioavailability varies widely from around 5% in the case of nisoldipine to 60-80% in the case of amlodipine [21].

A recent review of 236 articles showed that the concomitant administration of CCBs (nifedipine, amlodipine, nicardipine, felodipine, nisoldipine, barnidipine, isradipine, verapamil, diltiazem) with grapefruit products (mainly, grapefruit juice) causes an increase of oral bioavailability of these medicines and risk of side effects (edem, flush, hypotension). The increase in the plasma concentration of CCBs are related to the down-regulation of intestinal CYP3A4 by grapefruit products (fresh fruit juice, frozen concentrate, whole fruit) [8]. The effects are more pronounced with the CCBs that have lower bioavailability (nimodipine, nisoldipine). Thus, intake of grapefruit juice (200-600 mL qd or bid for 2-3 days), increases the exposure to nisoldipine by 85% [5]. A single glass (200-250 mL) of regular-strength grapefruit juice can produce a several-fold increase in AUC and *C*_max_ of felodipine, although with considerable interindividual variability (Table 1). Also, an important increase in felodipine AUC and *C*_max_, respectively was noticed in the case of grapefruit juice consumption for several days. The changes of felodipine plasma concentrations are correlated with an increasing frequency of vasodilation-related side effects and pronounced decrease of blood pressure [6]. The cumulative effect of grapefruit juice can be related to the decrease of CYP3A4 by a post-transcriptional mechanism that involve an accelerated CYP3A4 degradation and the restoration of enzyme activity requires* de novo* synthesis [6]. Amlodipine and nifedipine that have a better bioavailability are less affected by the coadministration of grapefruit juice. Also, for nondihydropyridine calcium channel blockers, diltiazem and verapamil, only slight interactions have been described [22]. In the case of verapamil even its bioavailability is low, the magnitude of interaction was slight and mainly with grapefruit juice in multiple doses and long-acting verapamil. Besides, the biotransformation of verapamil is mediated via both CYP3A4 and CYP1A2 enzymes [23]. Flavonoids (naringin) and mainly furanocoumarins (bergamottin, 6',7'-dihydrobergamottin) are the major components responsible for CYP3A4 inhibitory effects of grapefruit [24]. Sevilla orange juice (bitter orange, sour orange) that contain the same furanocoumarins increase the systemic exposure to felodipine at coadministration [19]. Furanocoumarins strongly inhibit intestinal metabolism of drugs by covalent binding of CYP3A4 until new active enzymes are synthesized (around 24 hours); the major changes noted are increased plasma concentrations of administered drugs without the alteration of t_1/2_, which are heavily dependent on the hepatic metabolism, tissue distribution and renal elimination. The risk is significant when interval between grapefruit consumption and the drug intake is less than 4 hours. However, even a 10-hour interval showed an interaction risk of around 59%, while for 24 hours, the risk diminished to 25%. A 3-day interval between grapefruit intake and drugs intake completely removes the risks, this being the period for a complete renewal of intestinal CYP3A4 activity. Patients over 70 years of age, with multiple medications, who consume grapefruits, are more likely to develop serious or fatal interactions [8].

Nifedipine and verapamil have been reported to interact with* Hypericum perforatum* [25]. Concomitant intake of St. John's wort (900 mg extract/day, for more than 10 days) increases the systemic clearance of nifedipine and verapamil via the induction of intestinal and hepatic CYP3A4 activity. Hyperforin, a prenylated phloroglucinol derivative, is a main compound of St. John's wort plants. It showed strong agonist properties for human PXR (Ki=27 nM) which would explain the St. John's wort influence on the activity of the CYP3A4 enzyme and P-gp transporters [5].

The simultaneous ingestion of Ginkgo extract (240 mg/day) and nifedipine (10 mg/day) do not significantly affect any of the pharmacokinetic parameters of nifedipine or its metabolite (dehydronifedipine). Only some subjects experienced a 2-fold increase in C_max_ value of nifedipine and they experienced longer-lasting headaches (compared to the control group), dizziness and hot flushes (Table 1). At the same time, the heart rate is faster after the combined administration of nifedipine with ginkgo extract than with single dose. A pharmacodynamic interaction is presumed to interfere, but the mechanism is unknown [26]. It is recommended that nifedipine and other similar CCBs should not be administered with* Ginkgo* extracts, a careful monitoring being required for concomitant use in humans.

### 2.3. Direct Renin Inhibitors

The renin inhibitors are meaningful agents in the treatment of essential hypertension. They target the renin-angiotensin-aldosteron system, that plays a key role in the regulation of vascular and cardiac functions. Aliskiren is a first-in-class oral renin inhibitor and only agent approved by FDA in 2007, which provided an antihypertensive efficacy comparable to that of angiotensin receptor blockers [27, 28]. It exhibits a low bioavailability being slightly metabolized by CYP3A4 but the extent of metabolism is unknown. Besides, aliskiren is substrate of uptake (OATP2B1) and efflux (P-gp) transporters [29].

Regular consumption of apple and orange juices can significantly reduce the plasma concentrations of aliskiren (Table 1). In line with the reduction of aliskiren absorption from gastrointestinal tract, an attenuation of its antihypertensive effect was noticed. The most likely mechanism of these interactions involves the inhibition of the OATP2B1-mediated intestinal absorption of aliskiren [30]. Certain flavonoids of fruit juices such as hesperidin, tangeritin and nobiletin from orange juice and phlorodzin, quercetin and kaempferol from apple juice, have been shown to inhibit OATP2B1-mediated uptake* in vitro *[19, 30]. Concomitant intake of aliskiren and orange or apple juice should be avoided [19].

Also, coadministration of a single dose (300 mL) or multiple doses of grapefruit juice (200 mL×3/day, 5 days) decreases systemic exposure to aliskiren. Rebello et al. [31] showed that the effect of single dose of grapefruit juice on aliskiren pharmacokinetic profile is not clinically relevant. It is mediated via inhibition of the intestinal OATP1A2 transporter. Using* in vitro *experiments, the same group of researchers pointed out that aliskiren is a likely substrate for OATP1A2 and naringin, a major flavanone of grapefruit juice, reduces the uptake of aliskiren in OATP1A2-expressing cells (IC_50_=75 *μ*M) [31]. On the contrary, in a parallel study, Tapaninen et al. [29] reported significant clinical relevant effects of multiple doses of grapefruit juice on aliskiren pharmacokinetics (Table 1) and the involvement of OATP2B1 inhibition mechanism. In a review from 2017 about intestinal drugs interactions mediated by OATP transporters, the authors mention that aliskiren has proven to be substrate for both transporters but with moderate affinity for OATP2B1 (Km=72 mM) [32]. In addition, Shirasaka et al. [33] showed that naringin causes a significant decrease of OATP2B1 activity at the concentrations present in grapefruit juice. The different design of the study, dosage of grapefruit juice and aliskiren, physiological context, could influence and explain the discrepancies between the results. However, prolonged administration of high doses of grapefruit juice in patients with aliskiren should be avoided.

### 2.4. Non-Peptidergic Angiotensin II Receptor Blockers (ARBs, Sartans)

ARBs are prescribed mostly in the elderly patients with hypertension, type 2 diabetes, heart failure and left ventricular dysfunction. They antagonize angiotensin II-induced vasoconstriction, aldosterone and catecholamines release and hypertrophic response leading to the blood pressure lowering effects [34].

Losartan, the first orally available ARBs, is metabolized via CYP3A4 and CYP2C9 to E-3174, a pharmacologically active metabolite. Genomic variability in CYP2C9 isoenzyme may influence losartan metabolism [35]. The administration of silymarin (420 mg/day, 14 days), a well-known and valuable hepatoprotective drug obtained from milke thistle fruits (*Silybum marianum*), inhibits bioactivation of losartan. The magnitude of interaction is dependent of CYP2C9 genotype. In CYP2C9 wild-type subjects, silymarin reduces significantly the plasma concentration of E-3174 which could cause the decrease of clinical efficiency of losartan (Table 1) [36]. Also, Ginkgo and St. John's wort modulates the expression and genotype-dependent activity of CYP2C9 without any alteration in the case of poor metabolizers [5, 19].

### 2.5. Cardiac Inotropic Drugs

Digoxin is one of the most commonly indicated drugs in patients with atrial fibrillation and chronic congestive cardiac failure [37]. Its interactions are of interest due to its narrow therapeutic window. Digoxin is a P-gp substrate whose clearance is achieved by renal excretion that includes glomerular filtration and tubular secretion. Nearly all of the digoxin in the urine is excreted unchanged, with a small part as active metabolites [38].

Long-term administration of* Hypericum perforatum* extracts (over 10 days) decreases consistently the bioavailability of digoxin. The reduction in C_max_ and AUC of digoxin is supposed to reflect an influence on absorption or distribution, rather than metabolism. The interaction is apparently due to the intestinal induction of P-gp by St. John's Wort (Table 1). Hyperforin is the constituent of St. John's Wort, responsible for this interaction through its ligand properties for the nuclear PXR receptors that regulate the expression of P-pg. The degree of interaction of products based on St. John's wort and digoxin varies and it correlates with the level of hyperforin [25]. It appears that the significant plant-drug interactions with St John's wort have only occurred with extracts that result in an adequate hyperforin daily dose (at least > 3 mg) [39].

Siberian Ginseng (*Eleutherococcus senticosus*) significantly increases the serum digoxin level at the association. The mechanism of interaction is unknown. It seems that eleutherosides, bioactive phenylpropanoids of Siberian ginseng would be responsible. The cessation of administration of the herbal product causes, in time, the return to normal digoxin levels [40]. The increase in serum concentrations of medicines with low therapeutic index is problematic because it leads to the occurrence of toxicity (digoxin poisoning) that may endanger the patient's life. However, herbal products based on Siberian ginseng, and also on Asian ginseng (*Panax ginseng*), and Danshen (*Salvia miltiorrhiza*) have been shown to cross-react with digoxin monitoring assays, producing falsely elevated digoxin levels [41].

In human subjects, the coadministration of a standardized extract of ginkgo (27% flavonoids and 6% terpene lactones), 240 mg/day for 7 days with digoxin (0.25 mg/day) did not lead to significant differences in the control group, with respect to C_max_, T_max_, and AUC_0-1_. It can not be said whether there has been a simultaneous inhibition and induction of digoxin transport or renal filtration, which would have prevented a significant change in the digoxin bioavailability [7].

### 2.6. Antiarrhythmic Drugs

Grapefruit products (whole fruit, fresh juice or frozen concentrate) increase plasma concentrations and side effects of some antiarrhythmic drugs (amiodarone, quinidine, disopyramide and propafenone) by inhibiting their intestinal metabolism. The consumption of grapefruit products is not recommended in patients chronically treated with antiarrhythmics [8].

### 2.7. Oral Anticoagulants

Anticoagulant oral medicines include vitamin K antagonists (VKAs, coumarin anticoagulants) and direct-acting oral anticoagulants (DOACs). They are mainly used for the prevention of stroke in patients with atrial fibrillation and therapy of venous thromboembolism. VKAs act by interfering with vitamin K activation of clotting factors II, VII, IX and X, and also by inhibition of the regulatory anticoagulant protein C and S [42]. Warfarin is the most frequently prescribed VKAs and also frequently involved in many drug-drug and drug-plant products/drug-foods interactions with major clinical significance. Its narrow therapeutic window play a key role in the occurrence of these interactions.

58 different plant species can interact with warfarin in a clinical manner, and mainly* Hypericum perforatum*,* Allium sativum* (garlic),* Ginkgo biloba,* and* Panax ginseng*. 84% of the interactions are related to warfarin potentiation and 16% to warfarin inhibition. The larger risk occurs due to inappropriate or unattended use of plant products and consists of bleeding and haemorrhage. The effects occur by influencing the pharmacokinetics of warfarin, but also pharmacodynamicaly by influencing the platelet function, the coagulation cascade and fibrinolysis [8]. Warfarin is administered as a racemic mixture of S- and R-enantiomers. R-warfarin is metabolised under the action of CYP1A2 and CYP3A4, while S-warfarin predominantly via CYP2C19. The influence of CYP2C9 is particularly hazardous because S-warfarin is 3-5 times more potent than R-warfarin [43]. Pharmacotherapy of warfarin can be affected in many ways by concomitant use of plants that can cause unpredictable changes in the degree of therapeutic effectiveness experienced by patients [8].

*Hypericum perforatum* induces clearance of both forms, with a significant reduction in activity of racemate and a decrease in the international normalized ratio (INR) [25, 44]. At the same time, St. John's Wort causes a significant reduction in the plasma levels of phenprocoumon (a related coumarin anticoagulant) (Table 1). Hyperforin is a potent inducer of CYP1A2, CYP2C9, CYP2C19, CYP3A, CYP2E1 and P-gp activities in the liver and the small intestine. Enzymatic induction is dose-dependent [8].

Clinical trials of warfarin interactions with* Allium sativum* are inconclusive. Isolated case reports have revealed that the intake of garlic may increase INR and cause bleeding in warfarin-stabilized patients. The administration of six Kwai garlic tablets/day also led to a doubling of the INR value. In contrast, another controlled trial did not reveal any change in INR in warfarin-stabilized patients who received aged garlic extract (5 mL×2/day, 12 weeks) [45].* In vitro* assays have shown that various garlic products and some organosulfur compounds of garlic inhibit CYP2C9, CYP3A and CYPD6 isoenzymes. In animals, the inhibition of CYP2E1 and induction of CYP2C9 by garlic have been demonstrated. Also, the garlic constituent, allicin, and its degradation products possess antiplatelet effects as* in vitro* studies showed [45, 46]. In contrast, some clinical studies demonstrated that garlic had no effect on warfarin pharmacokinetics and pharmacodynamics [47]. However, the people with the wild-type* VKORC1* (vitamin K epoxide reductase subunit 1 gene) genotype exhibited a pharmacodynamic interaction with garlic [47]. It is possible that garlic interactions with warfarin may have a pharmacodynamic substrate, so a prudent approach is recommended in this association [45].

Coadministration of warfarin with* Ginkgo biloba *extracts showed mixed results. Few cases reported the bleeding or intracerebral hemorrhage at concomitant use of* Ginkgo* and warfarin. On the contrary, the administration of EGb761 (240 mg/day, 14 days) to male subjects (20-36 years), determined no change in the pharmacokinetic or pharmacodynamic parameters of warfarin (25 mg). Egb761 is a standardized extract in terpene lactones and flavonoids from* Ginkgo biloba* that are recommended for the therapy of neurosensory and cognitive deficits in the elderly and of peripheral vascular diseases. Ginkgo terpene lactones (ginkgolides) showed antiplatelet effects, and flavonoid fraction demonstrated* in vitro* inhibitory activity on CYP1A2, CYP2C9/19, CYP2D6 and CYP3A4 isoenzymes [48]. However,* in vitro* results can not easily be extrapolated to* in vivo* conditions or to other extracts, as the activity of ginkgo leaf constituents may not be relevant to the EGb761 extract administered* in vivo*. Amentoflavone, a biflavonoid that has been shown to be the most active inhibitor of CYP2C9, CYP2C19, CYP2D6 and CYP3A4 (IC_50_ = 0.035-4.8 mM) enzymes, is not actually found in EGb761. It is removed during the extraction process, as it lowers the bioavailability of simple flavonoids. It has also been shown that repeated doses of EGb761 do not result in a cumulative decrease in CYP2C9 enzyme activity [7]. However, it should be noted that the variability of ginkgo-based products is very high, and the use of non-standardized preparations may change the type of interaction. Concomitant administration of ginkgo with warfarin requires careful monitoring of INR and it is preferable to avoid association. Patients with cardiovascular and cerebrovascular conditions may use anticoagulants such as ticlopidine with ginkgo extract to reduce the risk of thrombotic events and increase microperfusion and cognitive performances. The treatment of healthy volunteers (20-29 years) with ginkgo extract (120 mg/day, 3 days) followed by ticlopidine administration (250 mg, single dose) and 40 mg ginkgo extract in the next day, did not alter C_max_ and AUC of ticlopidine, suggesting their potential for associated administration. However, studies conducted with low doses of the extract as well as short-term administration may not be appropriate to assess the risk of interaction in this case [7].

Although the studies showed contradictory results about the interactions between warfarin and ginseng-based products, their concomitant use is not recommended (Table 1). It appears that the administration of high doses of ginseng (over 1 g daily, prolonged use) could significantly change the pharmacological effect of warfarin [49].

Potential cranberry juice/products-warfarin drug interaction was described in several case reports. Coadministration of warfarin with large quantities of cranberry juice (more than 700 mL) or cranberry concentrate (1000 mg) for longer than several days or 3-4 weeks was associated with an increased INR and serious adverse effects, including fatal hemorrhage [8]. Some* in vitro* and animal studies suggested that cranberry exerts inhibitory effects on the CYP450 enzymes (CYP3A, CYP2C9) but the most of the clinical studies did not find a significant change in warfarin pharmacokinetics. A pharmacodynamic has also been suggested. It imply antiplatelet properties of some cranberry compounds (flavonoids, proanthocyanidins, and salicylic acid) [50]. Although, the exact mechanisms of this interaction are not well understood and there are certain inconsistency of clinical studies, the usage of cranberry products (mainly, high intake) in patients receiving warfarin should be avoided.

The consumption of grapefruit juice may affect the warfarin metabolism by inhibition of intestinal CYP2C9 and CYP3A4. Small clinical trials and case reports have shown an increase of INR (Table 1). Controlled studies are necessary to determine the magnitude of grapefruit juice (products) interaction with warfarin. At least for now it is more prudent to consider close follow-up and monitoring in patients on concurrent use of warfarin and grapefruit products or to avoid their coadminstration [8, 45, 51].

The ingestion of large amounts of green tea has been associated with a decrease in INR in patients receiving warfarin treatment. Although initially the interaction was explained by a pharmacodynamic mechanism based on the antagonism generated by the presence of vitamin K, however the amount is too low in green tea to produce this effect (1428 *μ*g/100 g of leaves and 0.03 *μ*g/100 g infusion) [45, 52]. However, pharmacokinetic data of warfarin are not available in the context of this association.

In the last 5 years, new direct anticoagulants have emerged: dabigatran, rivaroxaban, apixaban and edoxaban. The latter three agents are primarily metabolised by intestinal and liver CYP3A4 enzymes, and dabigatran is a P-gp substrate. Their metabolic profile increase the risk of interaction with grapefruit products in particular. At the same time, St. John's Wort may reduce their efficacy [8].

### 2.8. Statins

Statins are first choice drugs for the treatment of hypercholesterolaemia and prevention of coronary events, reducing significantly cardiovascular mortality.They inhibit hydroxymethylglutaryl-CoA (HMG-CoA) reductase enzyme, a key step in de* novo* synthesis of cholesterol. As a result, statins decrease cellular cholesterol content and the levels of atherogenic lipoproteins. In addition, they exert multiple beneficial pleiotropic effects that include the improvement of endothelial function, reduction of the inflammatory responses and of the smooth muscle cell proliferation [53, 54]. The currently available statins are predominantly metabolized by the CYP3A4 isoenzyme (simvastatin, atorvastatin, lovastatin) and CYP2C9 (fluvastatin). Pravastatin, pitavastatin and rosuvastatin do not undergo substantial metabolism by CYP450 pathway. Besides, simvastatin, atorvastatin and rosuvastatin are substrates for efflux ABCB1 transporter (P-gp) [53]. Also, all statins are substrates of OATP1B1, an uptake transporter expressed in hepatocyte membrane [54].

Controlled clinical trials have shown that concomitant treatment with St. John's wort extracts reduces plasma levels of simvastatin in healthy patients and those of atorvastatin in patients with hypercholesterolemia [55]. Effects are mediated by the induction of CYP3A4 isoenzyme and P-gp transporter and they are clinically relevant in the context of administration of St. John's wort products with high hyperforin content and prolonged use (at least 14 days). The combination with St. John's wort does not affect the clinical efficacy of pravastatin, which is not a substrate for CYP3A4 or P-gp [25, 55].

High daily intakes of grapefruit juice (which can also be the equivalent of 6 whole grapefruits/day) inhibits presystemic biotransformation of statins (lovastatin, simvastatin, atorvastatin) and increases consistently their systemic bioavailability (about by 13.5 times). A typical grapefruit juice intake (240 mL) increases moderately the systemic disposition of simvastatin (only by 3.6 times). The moment of ingestion grapefruit juice is also important. In the case of statins with short half-lives (simvastatin, lovastatin), the consumption of grapefruit juice in the morning will affect more pronounced their pharmacokinetics compared to the evening intake due to the fact that half-life of grapefruit juice effect is between 7 and 8 hours [56]. The inhibition of intestinal CYP3A4 by grapefruit juice is the main mechanism of interaction (Table 1). An interaction that leads to the increase of plasma levels of statins implies an increase in their adverse effects, particularly of rhabdomyolysis. However, in a recent study, Lee et al. [56] consider that the magnitude of the increased risk of rhabdomyolysis is uncertain and unlikely to exceed 1-2 per 100000 person years and the enhancing therapeutic efficiency of statins is more important. Authors suggest that moderate consumption of grapefruit juice should not be contraindicated in people taking statins. Perhaps a more cautious approach in this direction is desirable. The variable intake and variations of the grapefruit juice compounds, and also clinical status of patient may interfere, generating unpredictable interactions.

Concomitant administration of a single-dose of EGCG (300 mg), the main catechin of green tea, and rosuvastatin, decreases systemic exposure of this statin. However, multiple-dose pretreatment of EGCG (10 days) did not change the pharmacokinetics of rosuvastatin concomitantly administered with EGCG. A possible explanation is that a single dose of EGCG inhibits intestinal uptake transporters OATP2B1 or OATP1A2. Conversely, a prolonged treatment inhibits both absorption (intestinal transporters OATP2B1, OATP1A2) and elimination of rosuvastatin (hepatic uptake transporters OATP1B1 and OATP2B1). Also, it is plausible that the multiple-dose treatment with EGCG to cause an upregulation of OATP transporters in enterocytes and to increase the uptake of rosuvastatin [57]. It is interesting to see to what extent these pharmacokinetic data are valid for the use of green tea. The EGCG content varies largely in green tea infusion (2.3-203 mg/100 g infusion) as well as the daily intake of EGCG from the consumption of green tea infusions in EU (90-300 mg/day) [58]. The intake of EGCG is much higher in the case of high-level consumers (866 mg EGCG/day) or the use of food supplements with green tea catechins (5-1000 mg/day). Besides, the effects of pure EGCG may differ from green tea infusion or food supplements as respects the influence on drug transporters. Pharmacokinetics of catechins could be modified by the matrix in which they are present. The presence of other gallated catechins in green tea with similar EGCG properties, could enhance the effect of green tea on OATP drug transporters (OATP1A2, OATP1B1, OATP2B1) [57]. A relevant interaction characterized by a significant interindividual variability has been reported between green tea and simvastatin in Italian and Japanese subjects [59]. Prolonged use (14 days) of green tea increase the plasma concentrations of simvastatin. The effects were more pronounced in Japanese volunteers possibly in relation to the higher daily intake of tea catechins and EGCG than in the Italian study (638 mg and 322 mg, respectively versus 335 mg and 173 mg, respectively). Alongside with the inhibition of hepatic OATP1B1 transporter, other possible mechanisms that explain the green tea-simvastatin interaction may involve the inhibition of CYP3A4 metabolizing enzyme and/or P-gp efflux pump. However, the available data suggest a mild to moderate effect of green tea on CYP3A [59].

### 2.9. Other Cardiovascular Medicines

To the best of our knowledge, the clinical studies to evaluate pharmacokinetic interactions between plant products and cardiovascular drugs such as ACE inhibitors, diuretics and endothelin receptor antagonists, are lacking. However, taking into account the drug interaction information in the USPI of prescription drug products, the concurrent use of potent CYP3A4 inducers (*Hypericum perforatum*) with eplerenone, a selective aldosterone antagonist, should be avoided. St. John's wort can cause a decrease of eplerenone efficiency by enhancing the drug clearance [5, 60]. For the same reasons, the combined use of St.John's wort products with macitentan, an endothelin receptor antagonist which is approved for the treatment of cardiovascular diseases associated with chronic tissue endothelin system activation, is not recommended [61].

## 3. Drawbacks of Herbal Preparations Use

The main challenges associated with plant product use include scientific misidentification, product contamination and adulteration, mislabeling, variability in chemical composition, diversity of plant products and extraction methods, insufficient knowledge on phytocompounds pharmacokinetics, different regulatory systems for plant products and failure of disclosure on the part of patients.

The use of plant products in experimental or clinical studies to assess potential interactions should take into account the quality of plant extracts used in the study (composition, standardization, stability, content of specific components). Differences in the quality of plant extracts are responsible for divergent results obtained in clinical trials. In the case of gingko products, gold standard is EGb761, an extract characterized by following parameters drug to extract ratio (DER) = 35-67:1 (on the average 50:1), standardization in 22-27% flavonol glycosides, 5-7% terpene lactones (2.8-3.4% ginkgolides A, B, C and 2.6-3.2% bilobalid), less than 5 ppm ginkgolic acids. Its clinical efficacy in relieving symptoms associated with age-related cognitive decline, memory disorders, cerebral insufficiency and peripheral arterial disease is associated with doses of 120-240 mg/day. There are many* Ginkgo* supplements that contain diverse non-standardized hydroalcoholic extracts, without knowing their chemical composition. Numerous studies have also highlighted the adulteration of ginkgo products by the addition of pure flavonols and flavonol glycosides (rutin, quercetin, kaempferol) or extracts rich in flavonol-glycosides (*Fagopyrum esculentum*,* Sophora japonica*). The evaluation of 18 commercial ginkgo supplements from North America and Europe during 2015-2017 revealed that only 3 products contained genuine Ginkgo leaf extracts. Rutin, quercetin, kaempferol and* Sophora japonica* or green tea extracts have been identified as common adulterants. An investigation initiated by British Broadcasting Corporation and University of London's College of Pharmacy revealed that many of the* Ginkgo* supplements do not contain ginkgo extract or contain very low concentrations; 74% of the samples contain very high levels of rutin and/or quercetin. Also, the presence in large quantities of ginkgolic acids is dangerous due to their neurotoxic and allergenic properties. This type of contamination was reported in commercial samples from Europe, Japan, China, Australia, USA, Canada [107].

*Echinacea* is an important herbal medicines that is used in the prevention and treatment of upper respiratory tract infections. Modarai et al. [108] showed that the CYP3A4 inhibitory activity of* Echinacea* liquid preparations covaries with the total alklyalmide contens of extracts, with a > 150 fold difference between the most and least inhibitory product (IC_50_ = 12.7-1812 *μ*g/mL). The alkylamides are some of the bioactive immunomodulatory compounds of* Echinacea*. These constituents are present at widely different concentrations (1-1384.1 *μ*g/mL) depending on the species, part of the plant, type of extract and technique of extraction, nature of starting plant material (fresh/dry), the conditions of* Echinacea *products storage (temperature, time) [108, 109]. Similar comments can also be made in the case of St. John's wort products in terms of hyperforin and hypericins content, or that of green tea related to catechin and EGCG levels.The substitution of Koreen ginseng (*Panax ginseng*) with American ginseng (*P. quinquefolius*) or other inexpensive ginseng plants (*P. notoginseng*,* P. pseudoginseng* ssp.* japonicas*) or species (*Eleutherococcus senticosus*) may induce unpredicted therapeutic outcomes and interactions, due to their different chemical constituents [110, 111]. It is important for the raw plant material to specify the scientific botanical name of the species correctly identified, the parts of the plant used, the origin, and the processing method. For plant extracts information on the manufacturer, extractive technique, plant/extract ratio, chemical marker components should be provided [5, 19].

## 4. Conclusions and Future Perspectives

Although interactions between cardiovascular medicines and herbal products are increasingly reported or are increasingly suspected, they are very little anticipated in the current clinical routine. The magnitude of the use of herbal products is often unknown or there is a general appreciation that these products are safe and incapable of reacting with any medication. The development of a systematic method and rules is necessary to ameliorate the fundamental information deficiencies in the future assesment of the interactions between medicines and plant products/other xenobiotics. Many clinical trials are incomplete, with poor methodology. Inclusion in these studies for example of the typical eating or drinking habits, as well as the accuracy of pharmacokinetic-pharmacodynamic assessments is essential [8].

A better understanding of the molecular mechanisms involved in the interactions between plant products and cardiovascular medicines is necessary. From a clinician's point of view, a detailed health and diet history is essential to identify potential health problems to optimize prescription and dosage. Interactions identified by physicians should be reported to pharmacovigilance centers to gather information on such underestimated interactions. Anticipating the risk of interaction between drugs and herbal products requires a better understanding of the composition of the preparations. The main objective should be the development of standard manufacturing and control measures that ensure the quality and safety of plant products [8]. Genotyping of study participants would be helpful in identifying polymorphisms that can severely influence the clearance of synthetic drugs and plant metabolites. Then the intake of other herbal products, teas or fruit juices during clinical trials is problematic because they also contain a variety of metabolites that can interfere with the results or determine their own interactions [7].

A proper phytochemical characterization of plant extracts would be extremely valuable to minimize the risk of interaction as well as plant products variability. Further well-designed studies that include plant products with a well-established chemical composition, a robust pharmacokinetic analysis and the quantification of systemic exposure of product constituents are required to interpret the potential drug interactions and their clinical significance.

## Figures and Tables

**Table 1:** Clinically relevant pharmacokinetic plant-cardiovascular drugs interactions.

Cardiovascular medicines	Plant	Plant product	Dosage, Duration of administration	Plant bioactivity	Bioactive phytochemicals	Interaction	Putative mechanism	Ref.
*Calcium channel blockers*								

Felodipine	*Citrus × paradisi*	Grapefruit juice with 450 *μ*g naringin/mL (Old South grapefruit juice- Lykes Pasco Inc, USA)	200 mL, single dose	Cardioprotective Cholesterol-lowering effect Chemopreventive	Flavonoids Furanocoumarins	↑AUC, C_max_ of felodipine ↑antihypertensive effects	↓CYP3A4	[5, 62]
		Frozen juice concentrate (Miracle Mart, Canada)	200 mL (prepared by diluting 50 mL concentrate), single dose					[63]
		Regular-strength grapefruit juice (Everfresh Inc., Canada)	250 mL, single dose					[64]
		Intact grapefruit, Homogenized grapefruit segments Extract of segment-free parts (not available manufacturer)	Not available					[65]
	*Mentha piperita,* peppermint	Essential oil (not available manufacturer)	600 mg, single dose	Spasmolytic Carminative Antiemetic Expectorant	Menthol, Menthyl acetate	↑AUC, C_max_ of felodipine	↓CYP3A4	[66, 67]

Nifedipine	*Panax ginseng*, Korean ginseng	Not available	200 mg/day, 18 days	Adaptogenic	Ginsenosides	↑C_max_ of nifedipine (29%)	↓CYP3A4	[68]
	*Ginkgo biloba*	Not available	120 mg/day, 18 days	Neuroprotective Vascular effects	Ginkgolides Bilobalides Flavonoids	↑C_max_ of nifedipine (30%)	Unknown	[68]
		Ginkgolon-24, standardized extract to over 24% flavonoid glycosides, 6% terpene lactones, less than 1 ppm ginkgolic acids (Tokiwa Phytochemical Co. Ltd., Japan)	240 mg/day, single dose			↑C_max_, adverse reactions of nifedipine (only in some subjects)		[26]
	*Hypericum perforatum*, St. John's wort	Not available	900 mg/day, 18 days	Antidepressant	Hyperforin Hypericin Flavonoids	↓nifedipine blood concentration	↑CYP3A4	[67, 68]

Nisoldipine	*Citrus × paradisi*	Grapefruit juice (Everfresh Inc., Canada)	250 mL, single dose	Cardioprotective Cholesterol-lowering effect Chemopreventive	Flavonoids Furanocoumarins	↑bioavailabili ty of nisoldipine (85%)	↓CYP3A4	[5, 69]

Verapamil	*Hypericum perforatum*	Movina®, Standardized extract to hyperforin 3-6% (Boehringer Ingelheim, Sweden)	300 mg×3/day, 14 days	Antidepressant	Hyperforin Hypericin Flavonoids	↓AUC of R- and S-verapamil (78-80%)	↑CYP3A4	[67, 70]

*Cardiac inotropic drugs*								

Digoxin	*Hypericum perforatum*	Standardized extract LI 160 to 0.12-0.28% hypericin (Lichtwer Pharma AG, Germany)	900 mg/day, 10 days	Antidepressant	Hyperforin Hypericin Flavonoids	↓AUC_0-24_ of digoxin (25%) ↓C_max_ (33%)	↑P-gp	[70]
		Jarsin 300®, LI 160 extract (Lichtwer Pharma AG, Germany)	900 mg/day, 14 days			↓AUC of digoxin (18%)		[71]
		Standardized extract WS 5572 to 0.12-0.28% hypericin and 3-6% hyperforin (Dr. Willmar Schwabe Pharmaceuticals, Germany)	900 mg/day, 14 days			↓AUC of digoxin (23%)		[72]
		Remotiv, standardized extract ZE 117 to 0.2% hypericin and less than 0.5% hyperforin (Ewopharma, Czech Republic)	500 mg/day, 14 days			No change for AUC of digoxin		[73]
		Esbericum®, standardized extract to 1.47% hyperforin (Schape & Brummer, Germany)	240 mg/day, 11 days			↓AUC of digoxin (3%)		[74]
		Powder (not specified manufacturer)	2000 mg/day, 14 days			No change for AUC of digoxin		[73]
		Powder (not specified manufacturer)	2000 mg/day, 14 days			↓AUC of digoxin (18%)		[73]
		Powder (not specified manufacturer)	4000 mg/day, 14 days			↓AUC of digoxin (27%)		[75]
	*Thea sinensis*, green tea	Ggreen Tea catechin (Atlantic Essential Products, USA)	630 mg/day, pretreatment (13 days) and concomitant administration	Antioxidant Antiaging Neuroprotective Chemopreventive	Catechins (EC, EGCG)	↓AUC of digoxin (26-28%) ↓Cmax of digoxin (31-33%)	↑P-gp ↓digoxin uptake	[76]

*Beta-blockers*								

Atenolol	*Malus pumila*, apple	Apple juice (Martinelli's Gold Medal apple juice, USA)	1.2 L/day, in three phases of study	Antioxidant Anti-inflammatory Cardioprotective Anti-cancer	Flavonoids	↓AUC of atenolol (82%) ↓systemic exposure to atenolol	Unknown (possible mediated by OATP function and modulation of intestinal drug uptake)	[77]

Nadolol	*Thea sinensis*	Green tea beverage (Healthya, Japan)	700 mL/day, 14 days	CNS Stimulant Antioxidant Antiaging Neuroprotective Chemopreventive	Catechins (EGCG) Methylxanthines	↓AUC_0-48_ nadolol (85%) ↓ anti hypertensive effect	↓intestinal OATP1A2	[52, 78]

Talinolol	*Curcuma longa*, turmeric	Curcumin soft capsules (mixture of dimethoxy curcumin and bismethoxy curcumin) (ShenWei Pharmaceutical, China)	300 mg/day, 6 days	Anti-inflammatory Antioxidant Anti-carcinogenic Cholesterol-lowering effect	Curcuminoids	↓AUC, C_max_ of talinolol	↑P-gp	[79]
		Curcumin capsules (mixture of dimethoxy curcumin and bismethoxy curcumin) (Avmazon, USA)	1000 mg/day, 14 days			↑AUC, C_max_ of talinolol	↓intestinal P-gp in subjects with ABCB1 C3435T genotype	[18]
	*Ginkgo biloba*	Standardized extract (Rui Bang Pharmaceutical Company, China)	120 mg ×3/day, 14 days	Neuroprotective Vascular effects	Ginkgolides Bilobalides Flavonoids	↑AUC of talinolol (22-25%), ↑C_max_ of talinolol (36%)	↓intestinal P-gp	[16]
	*Citrus × paradisi*	Regular strength grapefruit juice (712 *μ*moL/L naringin; 492 *μ*mol/L dihydroxy-bergamottin and 45 *μ*moL/L bergamottin) (Paradiso- Succo di Pompelmo, Penny GmbH Deutschland, Germany)	300 mL (single dose); 900 mL/day, 6 days	Cardioprotective Cholesterol-lowering effect Chemopreventive	Flavonoids Furanocoumarins	↓AUC, C_max_ of talinolol (56-65%)	↓OATP	[80]
	*Schisandra chinensis*	Extract (16.85 mg deoxyschisandrin/tablet) (Oriental Pharmaceutical Company, China)	300 mg×2/day, 14 days	Adaptogenic Hepatoprotective	Lignans Flavonoids	↑AUC (47%) ↑C_max_ (51%)	↓P-gp	[16, 67]
	*Hypericum perforatum*	Jarsin 300®, LI 160 extract (Lichtwer Pharma AG, Germany)	900 mg/day, 12 days	Antidepressant	Hyperforin Hypericin Flavonoids	↓ talinolol concentration ↓AUC (31%)	↑P-gp	[67, 81]

*Direct renin inhibitors*								

Aliskiren	*Citrus × paradisi*	Normal-strength grapefruit juice (Valio Greipptäysmehu, Finland)	200 mL×3/day, 5 days	Cardioprotective Cholesterol-lowering effect Chemopreventive	Furanocoumarins Flavonoids	↓C_max_ (81%) ↓AUC_0-*∞*_ (61%) ↓t_1/2_	↓OATP2B1	[29]
		Grapefruit juice concentrate (Pfanner, Germany)	300 mL, single dose			↓Cmax (61%) ↓AUC (37%)	↓OATP1A2	[31]
	*Citrus × sinensis*, sweet orange	Concentrate normal-strength orange juice (Valio Greipptäysmehu, Finland)	200 mL×3/day, 5 days	Anti-inflammatory Cardioprotective Vascular effects Chlolesterol-lowering effect Antioxidant	Flavonoids	↓C_max_ (80%) ↓AUC_0-*∞*_ (62%)	↓OATP2B1	[30]
	*Malus pumila*, apple	Concentrate normal-strength apple juice (Valio Greipptäysmehu, Finland)	200 mL×3/day, 5 days	Antioxidant Anti-inflammatory Cardioprotective Anti-cancer	Flavonoids	↓C_max_ (84%) ↓AUC_0-*∞*_ (63%)	↓OATP2B1	[30]

*ARBs*								

Losartan	*Silybum marianum*, milk thistle	Silymarin (Madaus AG, Germany)	140 mg×3/day, 14 days	Hepatoprotective	Flavanolignans	*CYP2C9∗1/∗1* ↑AUC_0-24_ of losartan ↑AUC_0-*∞*_ losartan ↑C_max_ losartan CYP2C9∗1/∗1, CYP2C9∗1/∗3 ↓AUC_0-24_ of E-3174 ↓AUC_0-*∞*_ of E-3174 ↓C_max_ of E-3174	↓CYP2C9	[36]

*Oral anticoagulants*								

Phenprocoumon	*Hypericum perforatum*	Jarsin 300®, LI 160 extract (Lichtwer Pharma AG, Germany)	900 mg/day, single dose	Antidepressant	Hyperforin Hypericin Flavonoids	↓ AUC phenpro coumon (17%) ↓anticoagulant effect	↑CYP2C9 ↑CYY3A4	[67, 82]

Warfarin	*Hypericum perforatum*	Standardized extract equivalent to 1 g aerial parts, 0.825 mg hypericin and 12.5 mg hyperforin/tablet (Bioglan, Australia)	One tablet×3/day, 14 days	Antidepressant	Hyperforin Hypericin Flavonoids	↑warfarin clearance (27%) ↓INR	↑CYP1A2 ↑CYP3A4 ↑CYP2C19	[83, 84]
	*Panax ginseng*	Standardized extract G115 to ginsenosides 4% (Ginsana, Switzerland)	One capsule×3/day, 14 days	Adaptogenic	Ginsenosides	↓INR ↓anticoagulant effect	↑metabolism of warfarin (possible)	[49, 85, 86]
		Extract equivalent to 0.5 g root and 8.93 mg ginsenosides (ginsenoside Rg1)/capsule (Golden Glow, Australia)	2 capsules×3/ day, 7 days			No statistically significant changes of INR, PT and AUC values	Unknown	[83]
	*Panax quinquefolius* (American ginseng)	Root powder (Wisconsin Ginseng Board, USA)	1g×3/day, 21 days	Adaptogenic	Triterpenoids	↓INR ↓AUC, C_max_ of warfarin	Unknown	[67, 86]
	*Ginkgo biloba*	Tavonin™ – standardized extract EGb 761 (Schwabe Willmar GmbH&Co, Gemany)	2 tablets×3/day, 7 days	Neuroprotective Vascular effects	Bilobalid Ginkgolides Flavonoids	No effect on apparent clearance of warfarin enantiomers No effect on clotting status		[87]
		Various products based on *Ginkgo biloba *leaf extract (not specified manufacturer)	40 mg×3/day, 18 months; 40 mg×3-4/day, 6 months			↑effect of warfarin, bleeding effects	↓CYP2C9/C19, ↓CYP3A4 ↓CYP1A2	[88–91]
	*Thea sinensis*	Green tea beverage (not specified manufacturer)	0,5-1 gallon/day, 7 days	CNS Stimulant Antiaging Antioxidant Chemopreventive Neuroprotective	Catechins (EGCG) Methylxanthines	↓INR (3.79 vs. 1.37)	Unclear	[52, 92]
	*Citrus × paradisi*	Ready-to-drink grapefruit juice (President's Choice, Sunfresh Ltd, Canada)	1.5 L/day, 10 days	Cardioprotective Cholesterol-lowering effect Chemopreventive	Flavonoids Furanocoumarins	↑INR ↑effect of warfarin	↓CYP2C9, CYP3A4	[45, 93]
		Whole fruit (not specified manufacturer)	One fruit/day, 3 days					[51]
	*Glycine max (soybean)*	Soy milk (not available manufacturer)	480 mL/day, 4 weeks	Phytoestrogenic	Isoflavonoids	↓INR ↓effect warfarin	alterations of P-gp/OATP transporters ↓CYP2C9, CYP3A4	[45, 94]
	*Salvia miltiorrhiza* (Danshen)	Decoction	Dosage is not stated, 2 weeks	Antiplatelet	Tanshinone diterpenes	↑INR ↑effect of warfarin	↓ protein binding of warfarin ↑CYP1A2, CYP3A4	[45, 95]
		Herbal product (not specified manufacturer)	One month					[50, 96]

*Lipid lowering drugs*								

Atorvastatin	*Hypericum perforatum*	Movina®, Standardized extract to hyperforin 3-6% (Boehringer Ingelheim, Sweden)	300 mg×2/day, 28 days	Antidepressant	Hyperforin Hypericin Flavonoids	↓ C_max_, AUC ↓ atorvastatin efficiency	↑CYP3A4 ↑P-gp	[67, 97]
	*Citrus × paradisi*	Double strength grapefruit juice (Minute Maid frozen concentrated grapefruit juice, Coca Cola Foods, USA)	200 mL×3/day, 5 days	Cardioprotective Cholesterol-lowering effect Chemopreventive	Flavonoids Furanocoumarins	↑C_max_ (×2.6) ↑AUC_0-72_ (×3.3)	↓intestinal CYP3A4	[98]
		Florida grapefruit juice (not specified manufacturer)	300 mL/day, 90 days			↑serum levels of atorvastatin (19-26%) No adverse liver/muscle effects		[99]

Lovastatin	*Citrus × paradisi*	Double strength grapefruit juice (not available manufacturer)	200 mL×3/day, 3 days			↑C_max_ (×12) ↑AUC_0-12_ (×15)	↓intestinal CYP3A4	[100]

Pravastatin	*Hypericum perforatum*	TruNature®, Standardized extract to hypericin 0.3% (Leiner Health Products, USA)	300 mg×3/day, 14 days	Antidepressant	Hyperforin Hypericin Flavonoids	No significant effect on plasma concentrations	-	[101]

Rosuvastatin	EGCG (*Thea sinensis*)	Teavigo™ (Healthy Origin, USA)	300 mg/day, 12 days	CNS Stimulant Antiaging Antioxidant Chemopreventive Neuroprotective	Catechins	↓exposure to rosuvastatin (19%) at single dose of EGCG	↓intestinal OATP1A2/ OATB2P1	[57]
	*Hypericum perforatum*	Herbal supplement with 300 mg St. John's wort/capsule (not specified manufacturer)	2 capsules/day	Antidepressant	Hyperforin Hypericin Flavonoids	↓ rosuvastatin efficiency	↑P-gp	[67, 102]

Simvastatin	*Hypericum perforatum*	TruNature®, Standardized extract to hypericin 0.3% (Leiner Health Products, USA)	300 mg×3/day, 14 days	Antidepressant	Hyperforin Hypericin Flavonoids	↓AUC simvastatin ↓C_max_ simvastatin	↑CYP3A4 ↑P-gp	[101]
		Movina®, Standardized extract to hyperforin 3-6% (Boehringer Ingelheim, Sweden)	300 mg×3/day, 28 days			↑LDLc ↓ simvastatin efficiency		[103]
	*Citrus × paradisi*	Double strength grapefruit juice (Minute Maid frozen concentrated grapefruit juice, Coca Cola Foods, USA)	200 mL×3/day, 3 days	Cardioprotective Cholesterol-lowering effect Chemopreventive	Flavonoids Furanocoumarins	↑AUC_0-*∞*_ simvastatin (×16) ↑C_max_ simvastatin (×9)	↓intestinal CYP3A4	[5, 104]
		Normal-strength (Valio Ltd., Finland)	200 mL/day, 3 days			↑AUC_0-24_ (×3.6) ↑Cmax (3.9)		[105]
		Standard grapefruit juice (Morinaga, Japan)	200 mL×2/day, 2 days			↑AUC of simvastatin (×1.7)		[106]

AUC, area under the concentration-time curve; C_max_, maximum plasma concentration; EC, epicatechin; EGCG, epigallocatechin 3-gallate; INR, international normalized ratio; LDLc, low-density lipoprotein (LDL) cholesterol; PT, prothrombin time.

## References

[1] Na D. H., Ji H. Y., Park E. J., Kim M. S., Liu K., Lee H. S. (2011). Evaluation of metabolism-mediated herb-drug interactions. *Archives of Pharmacal Research*.

[2] Izzo A. A., Hoon-Kim S., Radhakrishnan R., Williamson E. M. (2016). A critical approach to evaluating clinical efficacy, adverse events and drug interactions of herbal remedies. *Phytotherapy Research*.

[3] Awortwe C., Bruckmueller H., Cascorbi I. (2019). Interaction of herbal products with prescribed medications: A systematic review and meta-analysis. *Pharmacological Research*.

[4] Tsai H.-H., Lin H.-W., Simon Pickard A., Tsai H.-Y., Mahady G. B. (2012). Evaluation of documented drug interactions and contraindications associated with herbs and dietary supplements: a systematic literature review. *International Journal of Clinical Practice*.

[5] Grimstein M., Huang S. (2018). A regulatory science viewpoint on botanical–drug interactions. *Journal of Food and Drug Analysis*.

[6] Bailey D. G., Malcolm J., Arnold O., Spence J. D. (1998). Grapefruit juice-drug interactions. *British Journal of Clinical Pharmacology*.

[7] Unger M. (2013). Pharmacokinetic drug interactions involving *Ginkgo biloba*. *Drug Metabolism Reviews*.

[8] Mouly S., Lloret-Linares C., Sellier P.-O., Sene D., Bergmann J.-F. (2017). Is the clinical relevance of drug-food and drug-herb interactions limited to grapefruit juice and Saint-John's Wort?. *Pharmacological Research*.

[9] Mohamed M. E., Frye R. F. (2011). Effects of herbal supplements on drug glucuronidation. Review of clinical, animal, and in vitro studies. *Planta Medica*.

[10] Rowland A., Miners J. O., Mackenzie P. I. (2013). The UDP-glucuronosyltransferases: their role in drug metabolism and detoxification. *The International Journal of Biochemistry & Cell Biology*.

[11] Williams J. A., Hyland R., Jones B. C. (2004). Drug-drug interactions for UDP-glucuronosyltransferase substrates: A pharmacokinetic explanation for typically observed low exposure (AUC 1/AUC) ratios. *Drug Metabolism and Disposition*.

[12] Won C. S., Oberlies N. H., Paine M. F. (2012). Mechanisms underlying food-drug interactions: Inhibition of intestinal metabolism and transport. *Pharmacology & Therapeutics*.

[13] Zha W. (2018). Transporter-mediated natural product–drug interactions for the treatment of cardiovascular diseases. *Journal of Food and Drug Analysis*.

[14] Ginghina C. (2016). *Compendium of Cardiovascular Diseases Therapy*.

[15] Matthaei J., Tzvetkov M. V., Gal V. (2016). Low heritability in pharmacokinetics of talinolol: A pharmacogenetic twin study on the heritability of the pharmacokinetics of talinolol, a putative probe drug of MDR1 and other membrane transporters. *Genome Medicine*.

[16] Fan L., Mao X., Tao G. (2009). Effect of *Schisandra chinensis* extract and *Ginkgo biloba* extract on the pharmacokinetics of talinolol in healthy volunteers. *Xenobiotica*.

[17] Juan H., Jing T., Wan-Hua Y., Juan S., Xiao-Lei L., Wen-Xing P. (2013). P-gp induction by curcumin: An effective antidotal pathway. *Journal of Bioequivalence and Bioavailability*.

[18] He X., Mo L., Li Z., Tan Z., Chen Y., Ouyang D. (2012). Effects of curcumin on the pharmacokinetics of talinolol in human with ABCB1 polymorphism. *Xenobiotica*.

[19] Chen M., Zhou S., Fabriaga E., Zhang P., Zhou Q. (2018). Food-drug interactions precipitated by fruit juices other than grapefruit juice: An update review. *Journal of Food and Drug Analysis*.

[20] Bailey D. G. (2010). Fruit juice inhibition of uptake transport: a new type of food-drug interaction. *British Journal of Clinical Pharmacology*.

[21] Kannan S., Gogtay N., Thatte U. M. (2014). Interaction of calcium channel blockers and grapefruit juice in healthy adults. *Cochrane Database of Systematic Reviews*.

[22] Sica D. A. (2006). Interaction of grapefruit juice and calcium channel blocker. *American Journal of Hypertension*.

[23] Fuhr U., Müller-Peltzer H., Kern R. (2002). Effects of grapefruit juice and smoking on verapamil concentrations in steady state. *European Journal of Clinical Pharmacology*.

[24] Lian-Qing G., Fukuda K., Ohta T., Yamazoe Y. (2000). Role of furanocoumarin derivatives on grapefruit juice-mediated inhibition of human CYP3A activity. *Drug Metabolism and Disposition*.

[25] Russo E., Scicchitano F., Whalley B. J. (2014). Hypericum perforatum: Pharmacokinetic, mechanism of action, tolerability, and clinical drug-drug interactions. *Phytotherapy Research*.

[26] Yoshioka M., Ohnishi N., Koishi T. (2004). Studies on interactions between functional foods or dietary supplements and medicines. IV. Effects of Ginkgo biloba leaf extract on the pharmacokinetics and pharmacodynamics of nifedipine in healthy volunteers. *Biological & Pharmaceutical Bulletin*.

[27] Wal P., Wal A., Rai A., Dixit A. (2011). Aliskiren: an orally active renin inhibitor. *Journal of Pharmacy and Bioallied Sciences*.

[28] Gradman A. H., Schmieder R. E., Lins R. L., Nussberger J., Chiang Y., Bedigian M. P. (2005). Aliskiren, a novel orally effective renin inhibitor, provides dose-dependent antihypertensive efficacy and placebo-like tolerability in hypertensive patients. *Circulation*.

[29] Tapaninen T., Neuvonen P. J., Niemi M. (2010). Grapefruit juice greatly reduces the plasma concentrations of the OATP2B1 and CYP3A4 substrate aliskiren. *Clinical Pharmacology & Therapeutics*.

[30] Tapaninen T., Neuvonen P. J., Niemi M. (2011). Orange and apple juice greatly reduce the plasma concentrations of the OATP2B1 substrate aliskiren. *British Journal of Clinical Pharmacology*.

[31] Rebello S., Zhao S., Hariry S. (2012). Intestinal OATP1A2 inhibition as a potential mechanism for the effect of grapefruit juice on aliskiren pharmacokinetics in healthy subjects. *European Journal of Clinical Pharmacology*.

[32] Yu J., Zhou Z., Tay-Sontheimer J., Levy R. H., Ragueneau-Majlessi I. (2017). Intestinal drug interactions mediated by OATPs: a systematic review of preclinical and clinical findings. *Journal of Pharmaceutical Sciences*.

[33] Shirasaka Y., Shichiri M., Mori T., Nakanishi T., Tamai I. (2013). Major active components in grapefruit, orange, and apple juices responsible for OATP2B1-mediated drug interactions. *Journal of Pharmaceutical Sciences*.

[34] Barreras A., Gurk-Turner C. (2003). Angiotensin II receptor blockers. *Baylor University Medical Center Proceedings*.

[35] Whirl-Carrillo M., McDonagh E. M., Hebert J. M. (2012). Pharmacogenomics knowledge for personalized medicine. *Clinical Pharmacology & Therapeutics*.

[36] Han Y., Guo D., Chen Y., Chen Y., Tan Z.-R., Zhou H.-H. (2009). Effect of silymarin on the pharmacokinetics of losartan and its active metabolite E-3174 in healthy Chinese volunteers. *European Journal of Clinical Pharmacology*.

[37] Hasenfuss G., Teerlink J. R. (2011). Cardiac inotropes: current agents and future directions. *European Heart Journal*.

[38] Currie G. M., Wheat J. M., Kiat H. (2011). Pharmacokinetic considerations for digoxin in older people. *The Open Cardiovascular Medicine Journal *.

[39] Chrubasik-Hausmann S., Vlachojannis J., McLachlan A. J. (2019). Understanding drug interactions with St Johns wort (Hypericum perforatum L.): impact of hyperforin content. *Journal of Pharmacy and Pharmacology*.

[40] McRae S. (1996). Elevated serum digoxin levels in a patient taking digoxin and Siberian ginseng. *Canadian Medical Association Journal*.

[41] Ting L. S. L., Shalansky S. J., Neall E., Enson M. H. H. (2008). Use of dietary supplements by patients taking digoxin. *Canadian Journal of Hospitaly Pharmacy*.

[42] Zirlik A., Bode C. (2017). Vitamin K antagonists: relative strengths and weaknesses vs. direct oral anticoagulants for stroke prevention in patients with atrial fibrillation. *Journal of Thrombosis and Thrombolysis*.

[43] Leite P. M., Martins M. A. P., Castilho R. O. (2016). Review on mechanisms and interactions in concomitant use of herbs and warfarin therapy. *Biomedicine & Pharmacotherapy*.

[44] Cohen P. A., Ernst E. (2010). Safety of herbal supplements: a guide for cardiologists. *Cardiovascular Therapeutics*.

[45] Ge B., Zhang Z., Zuo Z. (2014). Updates on the clinical evidenced herb-warfarin interactions. *Evidence-Based Complementary and Alternative Medicine*.

[46] Foster B. C., Foster M. S., Vandenhoek S. (2001). An in vitro evaluation of human cytochrome P450 3A4 and P-glycoprotein inhibition by garlic. *Journal of Pharmacy & Pharmaceutical Sciences*.

[47] Mohammed Abdul M. I., Jiang X., Williams K. M. (2008). Pharmacodynamic interaction of warfarin with cranberry but not with garlic in healthy subjects. *British Journal of Pharmacology*.

[48] Gaudineau C., Beckerman R., Welbourn S., Auclair K. (2004). Inhibition of human P450 enzymes by multiple constituents of the *Ginkgo biloba* extract. *Biochemical and Biophysical Research Communications*.

[49] Milic N., Miloevic N., Golocorbin Kon S., Boic T., Abenavoli L., Borelli F. (2014). Warfarin interactions with medicinal herbs. *Natural Product Communications*.

[50] Nutescu E. A., Shapiro N. L., Ibrahim S., West P. (2006). Warfarin and its interactions with foods, herbs and other dietary supplements. *Expert Opinion on Drug Safety*.

[51] Bodiford A. B., Kessler F. O., Fermo J. D., Ragucci K. R. (2013). Elevated international normalized ratio with the consumption of grapefruit and use of warfarin. *Sage Open Medical Case Reports*.

[52] Werba J. P., Misaka S., Giroli M. G. (2018). Update of green tea interactions with cardiovascular drugs and putative mechanisms. *Journal of Food and Drug Analysis*.

[53] Sirtori C. R. (2014). The pharmacology of statins. *Pharmacological Research*.

[54] Chauvin B., Drouot S., Barrail-Tran A., Taburet A. (2013). Drug–drug interactions between HMG-CoA reductase inhibitors (statins) and antiviral protease inhibitors. *Clinical Pharmacokinetics*.

[55] Borrelli F., Izzo A. A. (2009). Herb-drug interactions with St John's Wort (hypericum perforatum): an update on clinical observations. *The AAPS Journal*.

[56] Lee J. W., Morris J. K., Wald N. J. (2016). Grapefruit juice and statins. *American Journal of Medicine*.

[57] Kim T., Ha N., Kim Y. (2017). Effect of epigallocatechin-3-gallate, major ingredient of green tea, on the pharmacokinetics of rosuvastatin in healthy volunteers. *Drug Design, Development and Therapy*.

[58] Younes M., Agget P., Aguillar F., Crebelli R., Dusemund B., Filipi M. (2018). Scientific opinion on the safety of green tea catechins-EFSA Panel on Food Additives and Nutrient Sources added to Food (ANS). *EFSA Journal*.

[59] Werba J. P., Misaka S., Giroli M. G. (2015). Overview of green tea interaction with cardiovascular drugs. *Current Pharmaceutical Design*.

[60] Baxter K. (2008). *Stockleys Drug Interactions*.

[61] European Medicines Agency-Opsumit https://www.ema.europa.eu/en/documents/product-information/opsumit-epar-product-information_en.pdf.

[62] Bailey D. G., Arnold J. M. O., Munoz C., Spence J. D. (1993). Grapefruit juice–felodipine interaction: Mechanism, predictability, and effect of naringin. *Clinical Pharmacology & Therapeutics*.

[63] Lundahl J., Regårdh C. G., Edgar B., Johnsson G. (1997). Effects of grapefruit juice ingestion - Pharmacokinetics and haemodynamics of intravenously and orally administered felodipine in healthy men. *European Journal of Clinical Pharmacology*.

[64] Bailey D. G., Arnold J. M. O., Bend J. R., Tran L. T., Spence J. D. (1995). Grapefruit juice-felodipine interaction: reproductibility and characterization with the extended release drug formulation. *British Journal of Clinical Pharmacology*.

[65] Bailey D. G., Dresser G. K., Kreeft J. H., Munoz C., Freeman D. J., Bend J. R. (2000). Grapefruit-felodipine interaction: effect of unprocessed fruit and probable active ingredients. *Pharmacokinetics and Drug Disposition*.

[66] Dresser G. K., Wacher V., Wong S., Wong H. T., Bailey D. G. (2002). Evaluation of peppermint oil and ascorbyl palmitate as inhibitors of cytochrome P4503A4 activity in vitro and in vivo. *Clinical Pharmacology & Therapeutics*.

[67] Izzo A. A. (2012). Interactions between herbs and conventional drugs: overview of the clinical data. *Medical Principles and Practice*.

[68] Smith M., Lin K., Zheng Y. (2001). An open trial of nifedipine-herb interactions: nifedipine with St. Johns wort, ginseng or Ginkgo biloba. *Clinical Pharmacology & Therapeutics*.

[69] Bailey D. G., Arnold J. M. O., Strong H. A., Munoz C., Spence J. D. (1993). Effect of grapefruit juice and naringin on nisoldipine pharmacokinetics. *Clinical Pharmacology & Therapeutics*.

[70] Johne A., Brockmöller J., Bauer S., Maurer A., Langhcinrich M., Roots I. (1999). Pharmacokinetic interaction of digoxin with an herbal extract from St John's wort (Hypericum perforatum). *Clinical Pharmacology & Therapeutics*.

[71] Dürr D., Stieger B., Kullak-Ublick G. A. (2000). St John's Wort induces intestinal P-glycoprotein/MDR1 and intestinal and hepatic CYP3A4. *Clinical Pharmacology & Therapeutics*.

[72] Gurley B. J., Swain A., Williams D. K., Barone G., Battu S. K. (2008). Gauging the clinical significance of P-glycoprotein-mediated herb-drug interactions: Comparative effects of St. John's wort, Echinacea, clarithromycin, and rifampin on digoxin pharmacokinetics. *Molecular Nutrition & Food Research*.

[73] Mueller S. C., Uehleke B., Woehling H. (2004). Effect of St John's wort dose and preparations on the pharmacokinetics of digoxin. *Clinical Pharmacology & Therapeutics*.

[74] Arold G., Donath F., Maurer A. (2005). No relevant interaction with alprazolam, caffeine, tolbutamide, and digoxin by treatment with a low-hyperforin St John's wort extract. *Planta Medica*.

[75] Mueller S. C., Majcher-Peszynska J., Mundkowski R. G. (2009). No clinically relevant CYP3A induction after St. John’s wort with low hyperforin content in healthy volunteers. *European Journal of Clinical Pharmacology*.

[76] Kim T., Shin K., Park J. (2018). Effect of green tea catechins on the pharmacokinetics of digoxin in humans. *Drug Design, Development and Therapy*.

[77] Jeon H., Jang I., Lee S. (2013). Apple juice greatly reduces systemic exposure to atenolol. *British Journal of Clinical Pharmacology*.

[78] Misaka S., Yatabe J., Müller F. (2014). Green tea ingestion greatly reduces plasma concentrations of nadolol in healthy subjects. *Clinical Pharmacology & Therapeutics*.

[79] Juan H., Terhaag B., Cong Z. (2007). Unexpected effect of concomitantly administered curcumin on the pharmacokinetics of talinolol in healthy Chinese volunteers. *European Journal of Clinical Pharmacology*.

[80] Schwarz U. I., Seemann D., Oertel R. (2005). Grapefruit juice ingestion significantly reduces talinolol bioavailability. *Clinical Pharmacology & Therapeutics*.

[81] Schwarz U. I., Hanso H., Oertel R. (2007). Induction of intestinal P-glycoprotein by St John's wort reduces the oral bioavailability of talinolol. *Clinical Pharmacology & Therapeutics*.

[82] Maurer A., Johne A., Bauer S., Brockmöller J., Donath F. (1999). Interaction of St. Johns wort extract with phenprocoumon. *European Journal of Clinical Pharmacology*.

[83] Jiang X., Williams K. M., Liauw W. S. (2004). Erratum: Effect of St John's wort and ginseng on the pharmacokinetics and pharmacodynamics of warfarin in healthy subjects (British Journal of Clinical Pharmacology (2004) 57 (592-599)). *British Journal of Clinical Pharmacology*.

[84] Yue Q., Bergquist C., Gerdén B. (2000). Safety of St John's wort (Hypericum perforatum). *The Lancet*.

[85] Janetzky K., Morreale A. P. (1997). Probable interaction between warfarin and ginseng. *American Journal of Health-System Pharmacy*.

[86] Yuan C.-S., Wei G., Dey L. (2004). Brief communication: American ginseng reduces warfarin's effect in healthy patients. A randomized, controlled trial. *Annals of Internal Medicine*.

[87] Jiang X., Williams K. M., Liauw W. S. (2005). Effect of ginkgo and ginger on the pharmacokinetics and pharmacodynamics of warfarin in healthy subjects. *British Journal of Clinical Pharmacology*.

[88] Rowin J., Lewis S. L. (1996). Spontaneous bilateral subdural hematomas associated with chronic Ginkgo biloba ingestion. *Neurology*.

[89] Vale S. (1998). Subarachnoid haemorrhage associated with Ginkgo biloba. *The Lancet*.

[90] Bent S., Goldberg H., Padula A., Avins A. L. (2005). Spontaneous bleeding associated with Ginkgo biloba: A case report and systematic review of the literature. *Journal of General Internal Medicine*.

[91] Oga E. F., Sekine S., Shitara Y., Horie T. (2016). Pharmacokinetic herb-drug interactions: insight into mechanisms and consequences. *European Journal of Drug Metabolism and Pharmacokinetics*.

[92] Taylor J. R., Wilt V. M. (1999). Probable antagonism of warfarin by green tea. *Annals of Pharmacotherapy*.

[93] Bartle W. R. (1999). Grapefruit juice might still be factor in warfarin response. *American Journal of Health-System Pharmacy*.

[94] Cambria-Kiely J. A. (2002). Effect of soy milk on warfarin efficacy. *Annals of Pharmacotherapy*.

[95] Izzat M. B., Yim A. P. C., El-Zufari M. H. (1998). A taste of Chinese medicine!. *The Annals of Thoracic Surgery*.

[96] Yu C. M., Chan J. C. N., Sanderson J. E. (1997). Chinese herbs and warfarin potentiation by ‘Danshen’. *Journal of Internal Medicine*.

[97] Andrén L., Andreasson Å., Eggertsen R. (2007). Interaction between a commercially available St. John's wort product (Movina) and atorvastatin in patients with hypercholesterolemia. *European Journal of Clinical Pharmacology*.

[98] Lilja J. J., Kivistö K. T., Neuvonen P. J. (1999). Grapefruit juice increases serum concentrations of atorvastatin and has no effect on pravastatin. *Clinical Pharmacology & Therapeutics*.

[99] Reddy P., Ellington D., Zhu Y. (2011). Serum concentrations and clinical effects of atorvastatin in patients taking grapefruit juice daily. *British Journal of Clinical Pharmacology*.

[100] Kantola T., Kivistö K. T., Neuvonen P. J. (1998). Grapefruit juice greatly increases serum concentrations of lovastatin and lovastatin acid. *Clinical Pharmacology & Therapeutics*.

[101] Sugimoto K., Ohmori M., Tsuruoka S., Nishki K., Kawaguchi A., Harada K. (2001). Different effects of St. Johns wort on the pharmacokinetics of simvastatin and pravastatin. *Clinical Pharmacology Therapeutics*.

[102] Gordon R. Y., Becker D. J., Rader D. J. (2009). Reduced efficacy of rosuvastatin by St. John's wort. *American Journal of Medicine*.

[103] Eggertsen R., Andreasson Å., Andrén L. (2007). Effects of treatment with a commercially available St John's Wort product (Movina®) on cholesterol levels in patients with hypercholesterolemia treated with simvastatin. *Scandinavian Journal of Primary Health Care*.

[104] Lilja J. J. (2001). *Effects of Grapefruit Juice on The Pharmacokinetics of Selected CYP3A4 Substrate Drugs [Academic Dissertation]*.

[105] Lilja J. J., Neuvonen M., Neuvonen P. J. (2004). Effects of regular consumption of grapefruit juice on the pharmacokinetics of simvastatin. *British Journal of Clinical Pharmacology*.

[106] Kurata A., Hagiwara H., Ohmomo T. (2012). Effects of grapefruit juice consumption on pharmacokinetics of low dose simvastatin: cross-over study with a review of the literature. *Medicinal Chemistry*.

[107] Gafner S. (2018). Adulteration of Ginkgo biloba leaf extract. *Botanical Adulterants Bulletin*.

[108] Modarai M., Yang M., Suter A., Kortenkamp A., Heinrich M. (2010). Metabolomic profiling of liquid Echinacea medicinal products with in vitro inhibitory effects on cytochrome 450 3A4 (CYP3A4). *Planta Medica*.

[109] Livesey J., Awang D., Arnason J., Letchamo W., Barrett M., Pennyroyal G. (1999). Effect of temperature on stability of marker constituents in Echinacea purpurea root formulations. *Phytomedicine*.

[110] Kim D.-H. (2012). Chemical diversity of *Panax ginseng*, *Panax quinquifolium*, and *Panax notoginseng*. *Journal of Ginseng Research*.

[111] Choi K.-T. (2008). Botanical characteristics, pharmacological effects and medicinal components of Korean Panax ginseng CA Meyer. *Acta Pharmacologica Sinica*.

